# Comparison of non-triggered magnetic resonance imaging and echocardiography for the assessment of left atrial volume and morphology

**DOI:** 10.1186/s12947-018-0134-y

**Published:** 2018-09-18

**Authors:** Nicoline W. E. van den Berg, Dean R. P. P. Chan Pin Yin, Wouter R. Berger, Jolien Neefs, Rianne H. A. C. M. De Bruin-Bon, Henk A. Marquering, Annelie Slaar, R. Nils Planken, Joris R. de Groot

**Affiliations:** 1 Amsterdam UMC, University of Amsterdam, Heart Center, Department of Clinical and Experimental Cardiology, Amsterdam Cardiovascular Sciences, Meibergdreef 9, Amsterdam, The Netherlands; 2 St. Antonius hospital, Department of cardiology and cardiothoracic surgery, Nieuwegein, The Netherlands; 3 Onze Lieve Vrouwe Hospital, Department of Cardiology, Amsterdam, The Netherlands; 4 Amsterdam UMC, University of Amsterdam, Department of Biomedical Engineering and Physics, Meibergdreef 9, Amsterdam, The Netherlands; 5 Amsterdam UMC, University of Amsterdam, Department of Radiology, Meibergdreef 9, Amsterdam, The Netherlands; 6 Westfriesgasthuis, Department of Radiology, Hoorn, The Netherlands

**Keywords:** Atrial fibrillation, Arrhythmias, Atrial remodeling, Transthoracic echocardiography, Magnetic resonance imaging, Atrial fibrillation ablation

## Abstract

**Background:**

Advanced atrial fibrillation (AF) patients have persistent AF, failed previous catheter ablation and/or an enlarged left atrium (LA), which is associated with a reduced success of AF ablation. Transthoracic echocardiography (TTE) and contrast enhanced magnetic resonance angiography (CE-MRA) are available to assess LA volume. However, it is unknown how these modalities relate in patients with advanced AF. We therefore compared the reproducibility of TTE and non-triggered CE-MRA in advanced AF patients and their ability to select patients with successful thoracoscopic AF ablation.

**Methods:**

Two independent observers measured LA volumes on 65 TTE and CE-MRA exams of advanced AF patients prior to AF ablation. Patients were followed after AF ablation with rhythm monitoring every 3 months for 1 year to determine AF recurrence. Inter-modality, inter- and intra-observer variability were determined using intraclass correlation coefficients (ICC). Receiver-operating characteristic (ROC) analysis was performed to determine sensitivity and specificity of TTE and CE-MRA volume and CE-MRA dimensions to identify patients with AF recurrence during follow-up.

**Results:**

LA enlargement ≥ 34 ml/m^2^ was present in 60% of the patients. CE-MRA and TTE demonstrated a good correlation for LA volume assessment (intraclass correlation, ICC = 0.86; *p* < 0.001) with larger volumes consistently measured by CE-MRA. Major discrepancies were mostly attributed to TTE acquisition. Craniocaudal enlargement discriminated patients with AF recurrence (AUC 0.67 [95% CI 0.55–0.85], *p* = 0.01).

**Conclusions:**

*Non-triggered* CE-MRA is a viable and reproducible 3D alternative for 2D TTE to assess LA volume in advanced AF patients. Craniocaudal enlargement was the only discriminator of AF recurrence after AF ablation.

## Background

Left atrial (LA) structural remodelling is an important prognostic factor in advanced atrial fibrillation (AF) patients and is clinically assessed by measuring the LA volume [1, 2]. LA volume has been shown a non-invasive predictor of outcome following thoracoscopic or catheter based pulmonary vein isolation (PVI) for AF ablation [3–5]. While PVI continues to emerge as effective strategy for AF treatment, adequate assessment of LA volume becomes increasingly important for the selection of patients for AF ablation, but may be hampered by a lack of sinus rhythm and asymmetrical enlargement in advanced AF patients [1, 2, 4].

Advanced AF patients, that is patients with persistent AF, previously failed catheter ablation or enlarged LA, are likely to have LA enlargement with an aberrant morphology and asymmetry. However, LA volume is standardly assessed with 2-dimensional (2D) transthoracic echocardiography (TTE), which is limited by 2D planes and mathematical assumptions. TTE is therefore unable to detect asymmetry or pronounced unidirectional atrial enlargment [6–8].

More recent 3D techniques such as 3D echocardiography, multi-detector computed tomography (CT) and cardiac magnetic resonance imaging (CMR), the reference standard for LA volume assessment, enable a reconstruction of the 3D LA cavity and may provide a better estimate of LA volume in advanced AF patients in particular [9–12]. *ECG-gated* CMR demonstrated a higher accuracy and reproducibility of LA volume assessment compared to TTE in mostly sinus rhythm or non-AF populations [12, 13].

Three dimensional echocardiography, CT and CMR techniques are not standardly available in the clinical setting, whereas the *non*-*ECG-gated* 3D contrast enhanced magnetic resonance angiography (CE-MRA) is by all means already widely used in patients with advanced AF. CE-MRA is implemented in the routine work-up for AF ablation therapy next to the standard 2D TTE in order to assess pulmonary vein (PV) anatomy^5^. CE-MRA has a shorter acquisition time compared to CMR and does not require the presence of sinus rhythm for *ECG-gating*, which has questionable added value in patients in AF.

CE-MRA may be an alternative for 2D TTE that enables a 3D reconstruction of the LA cavity and assessment of the asymmetrically enlarged LA of advanced AF patients undergoing AF ablation. However, there is limited data about CE-MRA in patients with advanced AF including patients with severely enlarged LA. Moreover, CE-MRA is not recommended by guidelines as limited normative data or evidence for a prognostic value or reverse remodelling after AF ablation in advanced AF patients is available [7, 12]. Finally, the effects of *non-ECG-gating* in the absence or presence of sinus rhythm remain unclear.

In this study, we tested the hypothesis that CE-MRA is as reliable as TTE to assess LA volume in patients being in sinus rhythm or AF. We aimed to determine the most reproducible modality for LA volume assessment and their ability to discriminate patients for thoracoscopic AF ablation in an advanced AF population.

## Methods

### Study design

We selected all consecutive patients scheduled for stand-alone thoracoscopic surgical ablation of AF in the Academic Medical Center (AMC), Amsterdam, between January and December 2013. Patients were identified from a prospectively entered registry. We performed a cross-sectional comparison of TTE and CE-MRA exams made during standard work-up for AF ablation surgery and followed patients for one year to determine success of the procedure. The Institutional Review Board waived written informed consent. The study was conducted in accordance with the Declaration of Helsinki.

### Study population

Patients undergoing thoracoscopic surgery in our centre have advanced AF, defined as persistent AF according to the ESC 2016 guidelines for the management of AF [1], enlarged left atria [6] or one or more previously failed catheter ablation^14^. Patients must have failed at least one class Ic or class III anti-arrhythmic drugs.

### Procedure and follow-up

The standard thoracoscopic surgical AF ablation procedure of the AMC was described in detail previously [14, 15]. All patients underwent radio frequent ablation of the left and right PV antrum (AtriCure Isolator™ Synergy™ bipolar RF ablation clamp). In persistent AF patients, additionally a superior line and trigone line were created (AtriCure Isolator™ Transpolar™pen). All ablation lines were tested for bidirectional block with epicardial electrodes connected to an EP system [16]. The four main ganglionated plexus were ablated, unless patients participated in the AFACT trial and were randomized to no GP ablation [14].

After AF ablation surgery, patients were followed in accordance with the 2012 HRS guidelines, which included a visit to the outpatient clinic every 3 months with ECG and Holter monitoring to determine AF recurrence in the first year. After an initial 3-month blanking period, all antiarrhythmic drugs (AADs) were discontinued. AF recurrence was defined according to current guidelines as a 30 s continuous rhythm registration of AF, or an ECG recording of an AF episode in patients not using AAD [5].

### Transthoracic echocardiography

All patients underwent 2D *triggered* TTE (Vivid 9, GE VingmedUltrasound AS, Horten, Norway). All TTE examinations were made specifically to determine LA volume and function during the work-up for AF ablation. Four- and two-chamber views were obtained by experienced cardiac echocardiographists according to the recommendations of the American Society of Echocardiography [6]. Recordings were made using a 1.6-MHz to 3.2-MHz transducer (System 9; GE Healthcare, Milwaukee, WI), digitized, and analysed offline with EchoPAC (GE Healtcare, 2015 General Electronic Co.).

**Fig. 1:**
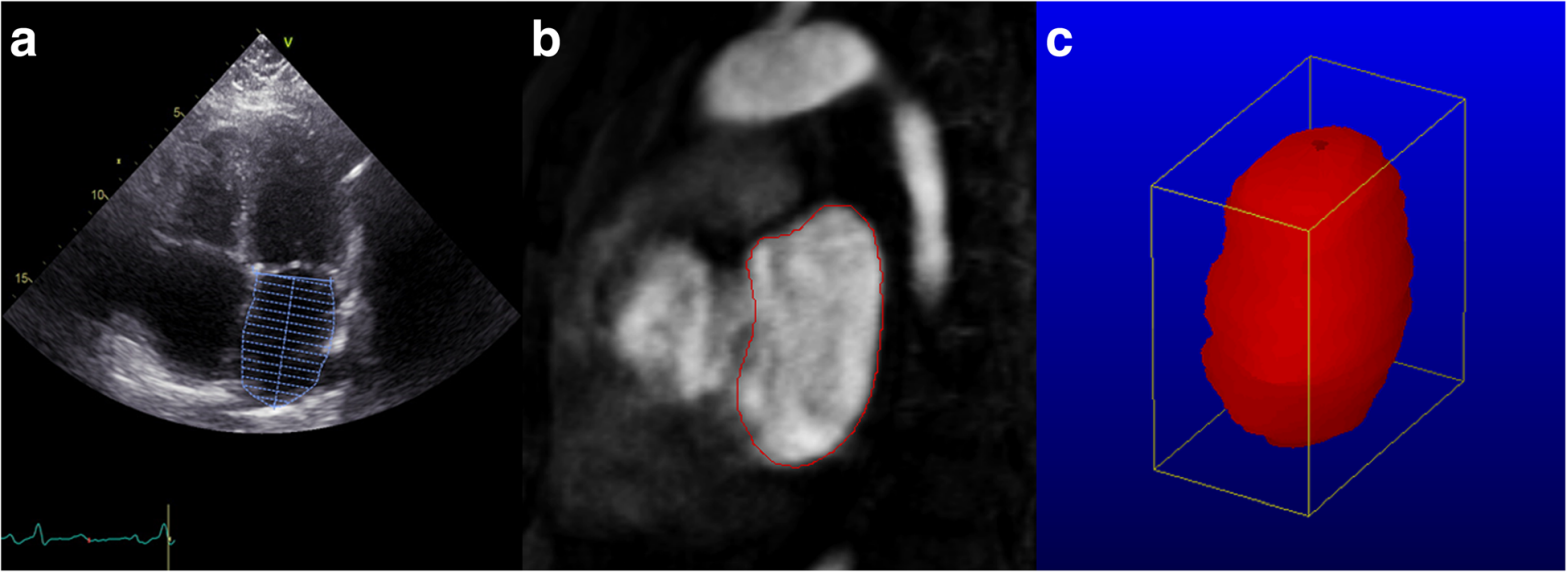
LA volume assessment with TTE and CE-MRA. **a** Example of a TTE apical 4-chamber view with delineation of the LA wall and exclusion of the LAA, PVs and area between the mitral valve annulus and mitral valve leaflets. Landmarks indicate the mitral valve level and LA length. **b** Example of a sagittal segment of CE-MRA with delineation of the LA wall. The atrioventricular groove and mitral valve were used as landmarks to separate the LA from the left ventricle. **c** 3-D reconstruction of the LA cavity, constructed from the CE-MRA segments

Maximum LA volume was defined as the tracing of the LA endocardial borders at the largest visual volume which is one or two frames prior to the opening of the mitral valve in the apical four- and two-chamber views and was calculated using the modified method of discs. In patients with AF at the time of TTE, we visually selected the frame with the largest volume from all available frames to eliminate the effect of reduced cardiac filling at short R-R intervals. We excluded the left atrial appendage (LAA), PVs and area between the mitral valve annulus and mitral valve leaflets from the LA volume determination (Fig. 1a) [6].

### Contrast enhanced magnetic resonance angiography

CE-MRA was performed using a 1.5 MRI scanner (Somatom Avanto MR B17 Siemens, Erlangen, Germany) with a 6 element body matrix surface coil. A standard dose of gadobutrol (0.1 mmol/kg of Gadovist; Bayer Vital GmbH, Leverkusen Germany) was injected by a power injector and through a 20-gauge plastic cannula placed in an antecubital vein for all examinations. For the MRI acquisition, a standard automated bolus injection of 0.1 mmol/kg body weight of gadobutrol (Gadovist, Bayer Schering Pharma, Germany) was used at a flow rate of 2 cc/sec, followed by 20 cc of saline flush, at the same rate. The MRI sequence with centric ordering of k-space was started manually as soon as the contrast agent was seen in the left atrium on the2D real-time fluoroscopy. We used a routine breath-hold technique and 2D contrast bolus tracking sequence to time the first pass CE-MRA acquisition. Imaging parameters were: 96 slices, FOV 500 × 344 mm, image resolution 1.3 × 1.3 × 1.3 mm, parallel imaging factor 2, image acquisition time 19 s.

LA volumes and dimensions were analysed offline using image analysis software (Medis Mass & Flow, Leiden, the Netherlands, version 2015-EXP). The LA was segmented using the CE-MRA images and LA volume was assessed by manual tracing of the endocardial borders of successive slides in the sagittal plane (Fig. 1b, c). The atrioventricular groove and mitral valve were used as landmarks to separate the LA from the left ventricle. The LAA and PVs were excluded, analogous to LA volume assessment with TTE. As an indication for LA enlargement in one direction, the maximal craniocaudal, anteroposterior and transversal dimensions were measured in the maximal sagittal and transversal planes respectively. 

### Repeated measurements

All patients underwent a CE-MRA and 2D TTE prior to AF ablation surgery. Two independent readers (N.v.d.B. and D.C.P.Y) retrospectively analysed all stored CE-MRA and TTE images to measure LA volume. A random sample of 25% was selected for repeated measurements by one observer to evaluate the intra-observer variability.

### Statistical analysis

Data are presented as frequencies, mean with SD or median with interquartiles as appropriate. TTE and CE-MRA LA volumes were defined as the mean of the volumes measured by two observers for each modality. Differences between LA volume on TTE and CE-MRA were evaluated using the Pearson correlation coefficient and intraclass correlation coefficients (ICC) with 95% confidence intervals (CI) as described by Shrout and Fleiss [17]. Consequently Bland-Altman analyses were constructed to demonstrate bias in units (ml) and as a percentage of the mean volume (%, bias/average of TTE and CE-MRA volume*100) and to assess the limits of agreement (LoA), defined as 1.96SD around the mean [18]. A Pearson correlation between the mean volume and volume difference was applied to determine the presence of proportional difference variability. Additionally, the Bland-Altman plots were used to identify the 10 most extreme outliers between TTE and CE-MRA volume measurements. The stored images of the most extreme outliers were retrospectively assessed and discussed for cues to explain the discrepancies. We determined the ICC of each modality for inter-observer and intra-observer variability. The analyses of inter-modality, inter-observer and intra-observer variability were performed for the entire study population and subsequently for stratified subgroups of patients known to be congruously in sinus rhythm or AF during both TTE and CE-MRA acquisitions.

Receiver-operator curve (ROC) analysis was performed with calculation of the area under the curve (AUC). The sensitivity and specificity of the cut-off value based on the Youden’s index was provided. ROC analyses were used to determine the discriminative value of LA volumes and CE-MRA dimensions to determine AF recurrence after thoracoscopic surgery.

Analyses of volume measurements were performed with both non-indexed volume measurements and volume measurements indexed for BSA. Because of evident similarities between the non-indexed and indexed measurements, we here reported the non-indexed measurements for technical comparisons and indexed values where relevant. For clinical comparisons, such as ROC analysis of outcome, the indexed volumes were used. All performed tests were two-sided and values of *P < 0.05* were considered statistically significant (IBM SPSS statistics version 24 and R, version 3.2.3).

## Results

### Patient population

We included a total of 65 out 68 patients who underwent thoracoscopic AF ablation in 2013: in one patient the echocardiogram could not be retrieved and in two patients a different MRI protocol was used for research purposes. Advanced AF patients consisted of 54% patients with persistent AF, 20% with previously failed catheter ablation and 60% with an enlarged LA ≥34 ml/m^2^. Five patients did not complete the study and could not be included in the outcome analysis: one thoracoscopic procedure was terminated preterm due to severe adhesions and 4 patients were lost-to-follow-up. Twenty-six (40%) patients had AF during TEE and 17 (35%) during CE-MRA. Fourteen patients had sinus rhythm during both examinations and 11 patients had AF during both TTE and CE-MRA. Sixty-three percent of the patients was free from AF after 1 year follow-up. Clinical characteristics are presented in Table 1.

**Table 1:** Patient characteristics

	65 patients
Sex, male, n (%)	46 (71)
Age, years (±SD)	60 ± 8
AF type, paroxysmal, n (%)	30 (46)
AF duration, years [IQ]	4 [1–35]
BMI, kg/m^2^ (±SD)	27 ± 4
BSA, m^2^ (±SD)	2.1 ± 0.2
NT-proBNP, pmol/l [IQ]	276 [50–3135]
Hypertension, n (%)	23 (35)
Diabetes Mellitus, n (%)	2 (3)
History myocardial infarction, n (%)	4 (6)
History PCI, n (%)	5 (8)
History congestive heart failure, n (%)	10 (15)
History PVI, n (%)	13 (20)
History of stroke, n (%)	5 (8)
Medication	
ß-blocker, n (%)	36 (54)
RAAS inhibition, n (%)	18 (28)
Digoxine, n (%)	9 (14)

Abbreviations: *AF* atrial fibrillation, *BMI* body mass index, *BSA* body surface area, *IQ* interquartiles, *PCI* percutaneous coronary intervention, *PVI* pulmonary vein isolation, *RAAS* renine angiotensin aldosterone system

### Comparison of volumes

**Fig. 2:**
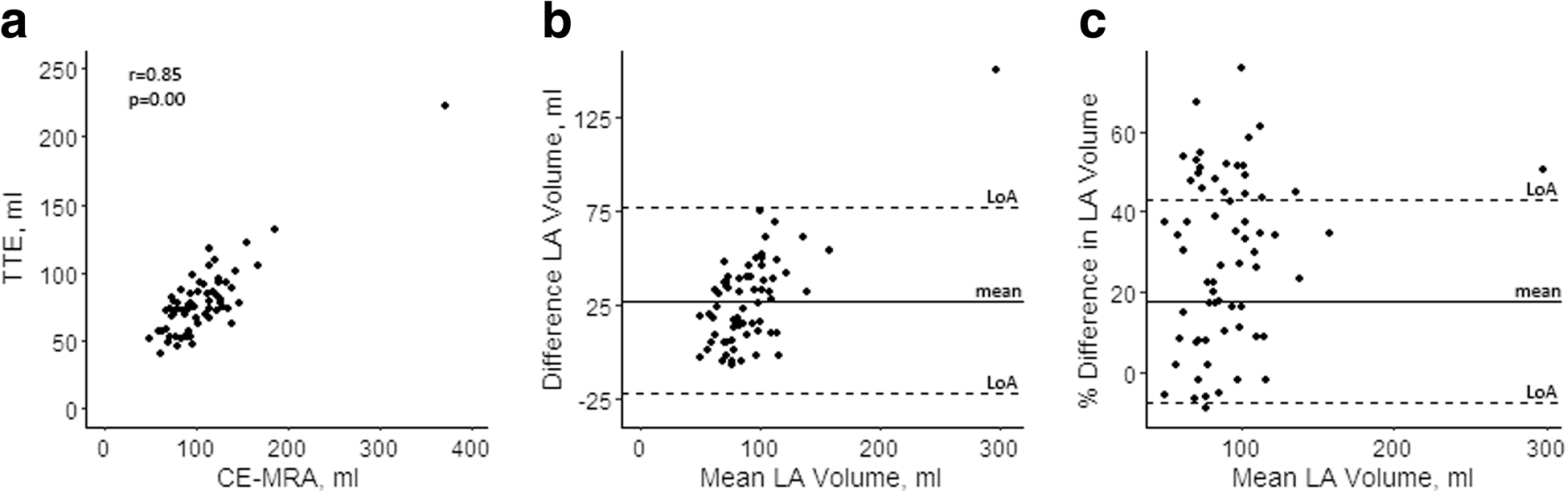
Relation between LA volume measurements by TTE and CE-MRA. **a** Correlation (Pearson r) between CE-MRA and TTE. **b** Bland-Altman plot of LA volume assessments by CE-MRA and TTE. **c** Bland-Altman plot of LA volume assessments by CE-MRA and TTE with the volume difference as a percentage of the mean volume. Abbreviations; CE-MRA, contrast enhanced magnetic resonance imaging; TTE, transthoracic echocardiography; LoA, limits of agreement; LA, left atrium

TTE and CE-MRA were performed within a median of five [IQ 1–19] days. Volume measurements differed significantly between TTE and CE-MRA, with larger LA volumes measured consistently by CE-MRA compared to TTE (TTE 78 ± 26 ml; CE-MRA 106 ± 44 ml; *p* < 0.0001)*(*Table 2*).* A strong correlation and agreement was found between TTE and CE-MRA (*r* = 0.85; *p* < 0.0001; ICC = 0.86; *p* < 0.001), but the Bland-Altman plots illustrate the wide limits of agreement (LoA) (− 22-77 ml)*(*Fig. 2*)*, indicating the large range of the systematic error. Moreover, there was an increase of the difference with increasing volumes (*r* = 0.70; *p* < 0.0001) (Fig. 2b)*.*

**Table 2:** LA volume measurements by TTE and CE-MRA

	CE-MRA	TTE	Bias (LoA)	Bias%	Pearson r	ICC (95% CI)
All patients *n* = 65						
LA volume, ml (±SD)	106 ± 44	78 ± 26	28 (− 22–77)	30%	0.85^a^	0.86 (0.77–0.91)_a_
LA volume index, ml/m^2^ (±SD)	51 ± 22	37 ± 13	14 (− 12–39)	32%	0.87^a^	0.86 (0.77–0.91)^a^
Sinus Rhythm *n* = 14						
LA volume, ml (±SD)	88 ± 24	67 ± 16	21 (−22–64)	27%	0.45	0.59 (−0.27–0.87)
LA volume index, ml/m^2^ (±SD)	45 ± 15	34 ± 9	11 (−13–35)	28%	0.57^a^	0.66 (− 0.05–0.89)^a^
Atrial Fibrillation *n* = 11						
LA volume, ml (±SD)	125 ± 28	85 ± 23	39 (13–65)	37%	0.88^a^	0.93 (0.74–0.98)_a_
LA volume index, ml/m^2^ (±SD)	58 ± 13	39 ± 9	19 (4–33)	38%	0.82^a^	0.87 (0.52–0.97)^a^
Discordant or unknown rhythm during CE-MRA *n* = 40						
LA volume, ml (±SD)	107 ± 50	80 ± 29	27 (−28–82)	29%	0.88^a^	0.87 (0.75–0.93)^a^
LA volume index, ml/m^2^ (±SD)	51 ± 26	38 ± 15	13 (−15–41)	29%	0.90^a^	0.88 (0.77–0.93)^a^

Abbreviations; *CE-MRA* contrast enhanced magnetic resonance imaging, *ICC* intraclass correlation coefficient, *LoA* limits of agreement, *TTE* transthoracic echocardiography

^a^Significant at *p* < 0.05

In patients with AF during both examinations, there was a statistically significant correlation and inter-modality agreement between TTE and CE-MRA. This was also the case in patients with sinus rhythm, however this did not reach statistical significance.

We excluded one extreme outlier *(*TTE: 222 ml, Z-score 5.5; MRI: 373 ml, Z-score: 6.1, Fig. 2*),* which was a female patient with 15 year history of AF, severe mitral valve disease, a history of non-obstructive cardiomyopathy and myocardial infarction. The exclusion of this outlier affected the correlation between TTE and CE-MRA, which became moderate and more strongly dependent on BSA (*r* = 0.69, *p* < 0.001; indexed *r* = 0.86, p < 0.001). The mean volume difference between TTE and CE-MRA was not importantly influenced by exclusion of this outlier (mean difference 26 ml) and the LoAs became narrower, but were still significantly large (LoA − 14-65 ml).

Post-hoc assessment of the 10 most extreme outliers between TTE and CE-MRA indicated foreshortening of TTE planes to be at least partly responsible for the difference in three cases. In another case, the PV were not adequately excluded on TTE and two TTE exams had poor image contrasts which complicated endocardial wall delineation and led to overestimation of the 2-chamber volume compared to the 4-chamber volume. In the remaining cases, no cause could be determined.

### Inter- and intra-observer variability

The inter- and intra-observer variability for both TTE and CE-MRA are shown in Tables 3 and 4*.* Both TTE and CE-MRA had high inter- and intra-observer agreements, which was slightly higher for CE-MRA demonstrated by the non-overlapping 95% CI in all patients.

**Table 3:** Inter- observer agreement

	Observer 1	Observer 2	Bias (LoA)	%Bias	Pearson r	ICC (95% CI)
All Patients						
TTE, ml (±SD)	79 ± 27	77 ± 27	2 (−22–24)	2%	0.91^a^	0.95 (0.92–0.97)
CE-MRA, ml (±SD)	111 ± 45	101 ± 43	10 (−6–25)	9%	0.98^a^	0.99 (0.99–0.99)
Sinus rhythm						
TTE, ml (±SD) (*n* = 41)	75 ± 19	72 ± 19	3 (−19–26)	5%	0.82^a^	0.90 (0.82–0.95)
CE-MRA, ml (±SD) (*n* = 15)	92 ± 23	84 ± 23	9 (−6–23)	10%	0.95^a^	0.97 (0.92–0.99)
Atrial Fibrillation						
TTE, ml (±SD) (*n* = 24)	8 ± 37	86 ± 35	-2 (−25–22)	2%	0.95^a^	0.97 (0.94–0.99)
CE-MRA, ml (±SD) (n = 14)	117 ± 35	110 ± 34	8 (−5–20)	7%	0.98^a^	0.99 (0.97–1.00)

Abbreviations: *CE-MRA* contrast enhanced magnetic resonance imaging, *ICC* intraclass correlation coefficient, *TTE* transthoracic echocardiography

^a^Significant at *p* < 0.01

**Table 4:** Intra-observer agreement

All Patients *n* = 16 (25%)	Read 1	Read 2	Bias (LoA)	%Bias	Pearson r	ICC (95% CI)
TTE, ml (±SD)	73 ± 22	76 ± 19	−3 (−18–12)	4%	0.94^a^	0.97 (0.90–0.99)
CE-MRA, ml (±SD)	104 ± 31	106 ± 30	−2 (−10–5)	2%	0.99^a^	1.00 (0.99–1.00)

Abbreviations: *CE-MRA* contrast enhanced magnetic resonance imaging, *ICC* intraclass correlation coefficient, *TTE* transthoracic echocardiography

^a^Significant at *p* < 0.01

### Discriminative value

The ROC analyses for the identification of patients with AF recurrence based on LA volume indexes and indexed CE-MRA dimensions are shown in Table 5. LA volumes determined by TTE and CE-MRA were similar for the discrimination between patients with AF recurrence and successful AF ablation, but both had a poor discriminative value (AUC 0.62 [95% CI 0.47–0.77], *p* = 0.08 and AUC 0.62 [0.48–0.77], p = 0.08 respectively). Only the craniocaudal dimension significantly and moderately discriminated patients with AF recurrence (AUC 0.67 [95% CI 0.55–0.85], *p* = 0.01).

**Table 5:** Area under the curve, cut-off values and corresponding sensitivity and specificity

	Failure *N* = 22	Success *N* = 38	*P*-value	AUC (95% CI)	Cut-off	Sensitivity	Specificity
TTE index, ml/m2 (±SD)	41 ± 19	35 ± 8	0.08	0.62 (0.47–0.77)	34	77%	53%
CE-MRA index, ml/m2 (±SD)	57 ± 33	47 ± 13	0.08	0.62 (0.48–0.77)	37	91%	32%
Craniocaudal axis index mm/m2, (±SD)	36 ± 6	32 ± 4	0.01	0.67 (0.55–0.85)^a^	32	81%	68%
Antroposterior axis index, mm/m2 (±SD)	19 ± 5	18 ± 3	0.38	0.53 (0.38–0.69)	NA	NA	NA
Transversal axis index mm/m2 (±SD)	36 ± 8	34 ± 6	0.48	0.52 (0.36–0.68)	NA	NA	NA

Abbreviations; *AUC* area under the curve, *CE-MRA* contrast enhanced magnetic resonance imaging, *TTE* transthoracic echocardiography

^a^Significant at *p* < 0.05

## Discussion

This study demonstrates a good correlation between TTE and CE-MRA in all patients with advanced AF, that is persistent AF, enlarged left atria, or previously failed catheter ablation. Both TTE and CE-MRA showed good inter- and intra-observer agreement, which was slightly higher for CE-MRA. CE-MRA was as reliable to select patients with advanced AF for successful AF ablation, but both modalities had only poor discriminative values. In this study, only unidirectional enlargement in the craniocaudal direction measured on CE-MRA was a discriminator to select patients with AF ablation success.

CE-MRA may be an already widely available 3D alternative for 2D TTE for the assessment of LA volume in advanced AF patients as we found a moderate to good correlation between TTE and CE-MRA with a systematic error in line with previous studies reporting larger volumes measured by CE-MRA compared to TTE [9, 11, 12]. We found a wide range of the systematic error between the two modalities, which was mostly attributed to errors in TTE acquisition. Exclusion of the PV, LAA and adequate delineation of the LA posterior wall and septum demonstrated to be especially challenging on TTE. In addition, acquisition of the correct planes may be dependent on the echocardiographist’s experience, despite extensive training. Incorrect acquisition may result in atrial foreshortening and may make endocardial wall delineation more difficult [19]. In line with these relative difficulties in TTE interpretation, we found that interpretation of LA volume on CE-MRA was slightly more reproducible than on TTE images as demonstrated by the non-overlapping 95% CI of the inter- and intra-observer ICC in all patients. For those differences that could not be explained by technical shortcomings or errors in acquisition, we speculate that they are the result of the inability of 2D TTE to adequately assess asymmetrical LAs. CE-MRA may further be advantageous over TTE as it enables a first assessment of the LAA for the presence of thrombi even before the patient is anaesthetized and undergoes TEE just prior to surgery [20]. CE-MRA during follow-up could subsequently be used to confirm complete closure of the LAA.

*Cine* CMR, CT and 3D echocardiography are 3D imaging techniques that are not standardly available in the clinical setting to assess LA volume. On the contrary, CE-MRA is already widely used and the absence of *ECG-gating* makes CE-MRA suitable for all advanced AF patients, including those in AF during the examination. However, the lack of *ECG-gating* may conversely result in blurred endocardial walls and does not allow a functional analysis. Moreover, LA volume may be underestimated if not measured at maximal size. Therefore, the poor and non-significant correlation between TTE and CE-MRA in patients in sinus rhythm can be the effect of selecting maximal LA volume on TTE, but not necessarily selecting maximal LA volume on CE-MRA. The resulting occasional smaller difference between TTE and CE-MRA may negatively have affected the correlation between the two modalities. This is irrespective of the consistently larger LA volume measured by the CE-MRA compared TTE.

We found that LA volume measured by both TTE and CE-MRA only modestly and non-significantly discriminated patients with success after AF ablation. Our study population included advanced AF patients with various comorbidities and thus biological variability. Interestingly, we found that LA enlargement in the craniocaudal direction was more discriminative for AF recurrence after thoracoscopic AF ablation than LA volume measurements. Although LA dimensions determined by CE-MRA are single dimensional measurements, our finding supports a more important role for asymmetrical or unidirectional enlargement in advanced AF patients. Various models have been designed to determine atrial asymmetry [21–25], with good predictive value for the outcome of AF ablation [22, 24, 25], of which one also indicated the craniocaudal direction as the most discriminative for the absence of AF after ablation [22]. So far, none of these models has reached clinical practice, likely in part due to the limited availability of the required 3D techniques and a lack of normative data. As such, CE-MRA as a 3D techniques may provide opportunities for future studies.

### Limitations

This study could not compare against a gold-standard method such as *cine* CE-MRA, which was not available due to the retrospective design of the study. Nevertheless, we compared TTE with *non-triggered* CE-MRA for LA volume assessment because these are two clinically relevant, but principally different modalities that are often obtained concurrently for patients undergoing AF ablation as demonstrated by our center. CE-MRA and TTE were taken 5 days apart as part of the clinical pre-ablation work-up, which may have had a small effect on LA volume measurements due to changes in fluid volume status.

We did not standardize the TTE measures for respirophasic changes. However, it is unlikely that the large difference between CE-MRA and TTE is fully explained by respiration [26]. Furthermore, we included TTEs and CE-MRAs of patients being in sinus rhythm as well as in AF as we aimed to study a population of advanced AF patients. Since the CE-MRA was non-triggered, a rhythm recording for CE-MRA was not always available and the subgroup analyses based on rhythm were limited to small patient numbers [8, 27].

## Conclusion

*Non-triggered* CE-MRA is a viable 3D alternative for 2D TTE to assess LA volume in advanced AF patients. CE-MRA, which consistently measured larger LA volumes, demonstrated to be slightly more reproducible then TTE and performed similar for discriminating advanced AF patients for success after thoracoscopic AF ablation. CE-MRA provides the opportunity to assess 3D asymmetrical remodeling supported by our finding that the craniocaudal axis measured by CE-MRA was the strongest and only significant discriminator of the absence of AF after AF ablation. Because of low discriminative values, LA volume measurements should not be used as the prime indicator to select candidates for AF ablation and future studies should focus on the incorporation of atrial asymmetry to improve LA characterization and patient stratification.
